# Modern Imaging Techniques in the Study and Disease Diagnosis of the Mammary Glands of Animals

**DOI:** 10.3390/vetsci10020083

**Published:** 2023-01-22

**Authors:** Mariana S. Barbagianni, Pagona G. Gouletsou

**Affiliations:** Faculty of Veterinary Science, School of Health Sciences, University of Thessaly, 43100 Karditsa, Greece

**Keywords:** computed tomography, contrast-enhanced, Doppler, magnetic resonance imaging, mammary gland, positron emission tomography, ultrasonography, three-dimensional, elastography

## Abstract

**Simple Summary:**

Various imaging techniques may be applied in research or clinical practice to evaluate pathological conditions of mammary glands and contribute to a diagnosis. This review describes imaging techniques that are used on farms and companion animals’ mammary glands. Specifically, computed tomography, positron emission tomography, magnetic resonance imaging, ultrasonographic techniques such as Doppler, contrast-enhanced and three-dimensional examination, and also elastography are presented. Furthermore, the relative bibliography is reviewed and discussed.

**Abstract:**

The study of the structure and function of the animals’ mammary glands is of key importance, as it reveals pathological processes at their onset, thus contributing to their immediate treatment. The most frequently studied mammary diseases are mastitis in cows and ewes and mammary tumours in dogs and cats. Various imaging techniques such as computed tomography, positron emission tomography, magnetic resonance imaging, and ultrasonographic techniques (Doppler, contrast-enchanced, three-dimensional and elastography) are available and can be applied in research or clinical practice in order to evaluate possible abnormalities in mammary glands, as well as to assist in the differential diagnosis. In this review, the above imaging technologies are described, and the perspectives of each method are highlighted. It is inferred that ultrasonographic modalities are the most frequently used imaging techniques for the diagnosis of clinical or subclinical mastitis and treatment guidance on a farm. In companion animals, a combination of imaging techniques should be applied for a more accurate diagnosis of mammary tumours. In any case, the confirmation of the diagnosis is provided by laboratory techniques.

## 1. Introduction

Various imaging techniques are available and can be applied in research or clinical practice, in order to evaluate possible abnormalities of mammary glands of domestic animals, as well as to assist in the differential diagnosis of masses within these organs [1,2]. Some of these techniques use radiation and rely on its spatial and temporal interaction with tissues in order to educe useful information from observations [3] whilst others use magnetic field and radio or high-frequency sound waves [4,5].

Anatomical features, structures or physiological processes can be assessed by employing different imaging methods [6,7]. The main methods used in the imaging of mammary glands of animals include computed tomography, positron emission tomography, magnetic resonance imaging, and ultrasonographic techniques (e.g., B-mode ultrasonography, Doppler ultrasonography, contrast-enchased ultrasonography, three-dimensional ultrasonography, and elastography) [2,4,5,8].

Computed tomography and magnetic resonance imaging provide information regarding the structure of the organs whilst positron emission tomography mainly provides information about their function [3]. The functional information derived from positron emission tomography images can be combined with anatomical information obtained from computed tomography images, thus in combination constitute the positron emission tomography/computed tomography scanning technique [3]. Doppler ultrasonography, contrast-enhanced ultrasonography, three-dimensional ultrasonography, and elastography are easy, fast, non-invasive diagnostic techniques, enabling immediate results for investigation of mammary gland abnormalities, although there are differences in the diagnostic efficacy between these modalities [5].

Although in human medicine clear diagnostic imaging guidelines have been proposed to investigate diseases of the mammary glands [9], in veterinary work, similar protocols have not been developed thus far. At the moment, only an algorithm has been proposed for the evaluation of sentinel lymph nodes in dogs with mammary carcinoma [10].

The objective of this review is to describe the process of modern imaging techniques that apply in the study and disease diagnosis of the mammary glands of animals and to highlight the perspectives of each method.

## 2. Computed Tomography

Computed tomography (CT) is a useful technique for the evaluation of anatomical structures, which employs high spatial resolution and moderate differentiation of tissue contrast [6]. For computed tomography imaging, an X-ray tube and X-ray detectors are necessary, which are positioned on opposite sides of a rotating ring. The X-ray source is rotated 360° around the animal, and data are obtained at each degree of rotation in order to calculate the degree of attenuation in small squares of the cross-section [11]. To construct a three-dimensional image, abundant two-dimensional X-ray images are taken around the axis of rotation [6]. X-rays pass through different tissues of the body of the animals and are attenuated at different rates, which depend on the density of different tissues [3]. In general, the equipment and scanning protocols that are being used for humans can be used, with some modifications, for animals [1]. Specifically, the doses of ionizing radiation required for the examination in animals are smaller than those used in humans, but not negligible [3]. However, in animals, the examination is always performed with the animal under general anaesthesia [12].

In farm animals, CT imaging has been reported only in cows. This technique has been used for the estimation of mammary parenchyma of heifers, showing a significant accuracy [13], as it excluded extra-parenchymal tissue (fat) more reliably than did physical dissection [11]. Moreover, CT has been employed for assessing mammary development as a means to predict future milk production [14]. However, there is no report yet on the use of CT for the diagnosis of pathological conditions of the mammary gland in farm animals.

In companion animals, CT has been used for mammary tumour diagnosis [15]. Indeed, the method has been suggested to be performed in all female dogs with malignant mammary tumours [2]. The CT is the examination of choice in cases of mammary tumours because it can provide thorough anatomical details, accurate depiction of calcification [15], visualization of soft tissue components of tumours, and high sensitivity in the detection of potential metastases [16]. In a study of sentinel lymph node metastasis in canine mammary tumours, contrast-enhanced CT imaging showed high sensitivity and specificity (87.5% and 89.3%, respectively) [17].

The advantages of CT imaging are the high spatial resolution (1 mm) and the accuracy of the images provided [6]. The disadvantages of the technique include the low soft tissue contrast, the little information regarding the function of the organs, the use of ionizing radiation [3], the reduced availability of equipment, and the high cost [14].

## 3. Positron Emission Tomography

Positron emission tomography (PET) is a relatively new imaging technique that is primarily applied in companion animals and can provide information about functional processes in three dimensions [8]. Intravenous administration of radiolabeled tracers is needed for this examination while specific positron emission tomography tracers are currently used for the evaluation of specific metabolic processes, e.g., glucose metabolism, oxygen utilization, or blood volume [7]. The tracer that is mainly used in veterinary work is the fluorine-18 fluorodeoxyglucose, which is a glucose analogue; in this, the hydroxyl group on the two-carbon of a glucose molecule is replaced by a radioactive fluoride isotope (18F) [8]. This tracer is also preferred in PET/CT scans in human oncology in order to discriminate between malignant and benign lesions [18], given that cancer cells have higher glucose metabolism and resort to the use of anaerobic glycolysis (‘Warburg phenomenon’) [19,20,21]. In fact, in human medicine, it has been found that PET/CT shows low sensitivity (52.2%,) but high specificity (91.6%) for axillary lymph node metastasis in patients with breast cancer [22]. During positron emission tomography imaging, blood samples should be collected repeatedly to evaluate radioactivity in the blood during the course of the study [23] while the time course of radioactivity within the target tissue (i.e., the mammary glands) is recorded by the scanner. The radioactive half-life period of isotopes used in this examination is short, which limits the availability of positron emission tomography imaging [3]. Although the technique has a lower spatial resolution compared to other imaging techniques, the use of combined PET/CT techniques can assist the investigator to overcome this factor [3]. In such a case, CT images must be taken immediately before PET scanning [8].

For performing the examination, animals should be fasted for 12 h [24], and a catheter should be placed intravenously for inducing anaesthesia and for injecting the tracers [3]. It is recommended to assess the animals continuously throughout the imaging procedure, by monitoring vital parametres (heart rate, respiration rate, body temperature, reflexes), as well as performing an electrocardiographic examination and pulse goniometry [24,25]. Occasionally, blood pressure, blood glucose concentration, and blood gases concentration should also be monitored [25].

Based on the current bibliography, in farm animals, the method has not been applied diagnostically so far. On the other hand, in companion animals, PET has been employed for the detection of mammary tumours [8,26]. Sanchez et al. [8] standardized the maximum uptake value of glucose (mean value: 1.1) and correlated it with tumour size and benign vs. malignant lesions. They found that the minimum tumour size needed to distinguish malignant lesions according to the maximum uptake value of glucose was 1.5 cm and a glucose uptake value >2 was 100% sensitive for malignancy, although no association between the maximum uptake value of glucose and histologic subtype or grade had been found.

The advantage of PET imaging is the collection of information through true functional imaging [8]. The disadvantages of this technique include the little anatomical information, the low sensitivity of the costly equipment, the reduced availability of equipment [24], and the necessity for using radioactive tracers. [3].

## 4. Magnetic Resonance Imaging

Magnetic resonance imaging (MRI) provides detailed images with better soft tissue contrast compared to CT [3]. The principles of the technology refer to using a powerful magnetic field in order to align the nuclear magnetization of atoms of hydrogen present in the water within the body of the animal examined [27]. Radiofrequency fields are used, with the aim to modify the alignment of this magnetization, which causes the hydrogen nuclei to produce a magnetic field that rotates and thus can be detected by the scanner. This signal can be modulated by further magnetic fields, which aim to produce adequate information for reconstructing an image of the animal under examination [3]. As magnetic resonance imaging explores only the morphological features of the organ under examination, the use of contrast-enhanced MRI in human medicine has been found to improve the specificity and sensitivity of diagnosis achieved with MRI alone [28,29].

MRI can be useful and provides important information for diagnosis in humans, in which mammography, ultrasonographic examination, or mammary biopsy could not lead to a diagnosis because it may show evidence of mammary tumours that had not been detected hitherto (i.e., by means of previous diagnostic techniques) [30].

During the performance of the technique, the animals examined must be under anaesthesia to allow easier manipulations and a speedier process. The mammary glands should be milked out (especially in ruminants) in order to decongest and visualize the mammary parenchyma more accurately with no compression [31]. In companion animals, the technique is performed in accordance with the principles of human mammography [4].

In farm animals, MRI has been used for the assessment of mammary glands. Specifically, Fowler et al. [31] and Stelwagen et al. [32], by this modality, estimated the volume of the mammary parenchyma in goats and heifers, respectively. Until now, MRI has not been used for the diagnosis of pathological conditions of the mammary gland in farm animals.

In companion animals, conventional static MRI was applied for the examination of the morphological characteristics of mammary tumours in dogs, such as their size, shape, and their tissue structure [4], while dynamic contrast-enhanced MRI providing information about the physiological properties of tumours [4]. Garamvolgyi et al. [4] used contrast-enhanced MRI for imaging the physiology of microcirculation, a technique that improved the diagnostic approach of canine mammary tumours. Later, Jaramillo-Chaustre et al. [33] reported the use of low-field equipment of MRI with administration of contrast agent to differentiate signal intensities of lymph nodes that collect lymph from tumour regions regarding healthy lymph nodes in dogs.

The advantages of magnetic resonance imaging include the high spatial (1 mm) resolution achieved during the examination, the excellent soft tissue contrast, and the lack of requirement to use X-rays or radioactive tracers [3]. Additionally, in human medicine, it has been found that MRI provides high sensitivity (94.6%) and specificity (74.2%) for breast cancer diagnosis, compared with ultrasound, or mammography. The sensitivity increases more with the combination of imaging techniques (ultrasonography, mammography, and MRI) [34]. The disadvantages of this technique include the need for high-cost equipment [3] and the reduced availability of equipment.

## 5. Ultrasonography

### 5.1. Doppler Examination

Doppler ultrasonography can be used for the estimation of blood flow into the mammary glands. Power Doppler imaging uses the amplitude of a Doppler shift to detect blood movement, but without information regarding its direction [35]. Colour Doppler images are generally combined with grayscale (B-mode) images to display duplex ultrasonography images while colour Doppler flow is the presentation of the velocity by colour scale and provides information regarding its localization with the identification of the type of vessel [5].

Usually, a B-mode ultrasonographic examination precedes the Doppler examination. The B-mode ultrasonographic examination provides information about the dimensions of the mammary glands, as well as about the echotexture (homogeneous or heterogeneous) and the echogenicity (hypo, hyperechoic, or mixed) of the parenchyma [5]. In a study of canine mastitis, it has been found that, during inflammation, the tissue had reduced echogenicity and that the distinct layers, characteristic of the normal mammary tissue, were lost [36]. In cattle mastitis, the sonographic image depends on the degree of structural changes that occur in the tissue [37]. A non-homogeneous hypoechoic pattern was imaged in the case of acute mastitis [38] while a hyperechoic pattern was imaged in chronic mastitis as a result of fibrosis [37]. However, criteria allowing the differentiation between inflammation and neoplasia by ultrasonographic examination are not provided by current literature. Inflammation is very common in mammary neoplasia, especially during the prolonged process of tumour, and also, in clinical practice, mastitis may be indistinguishable from a mammary tumour with present inflammation [39], as has been reported in human medicine [40].

**Figure 1:**
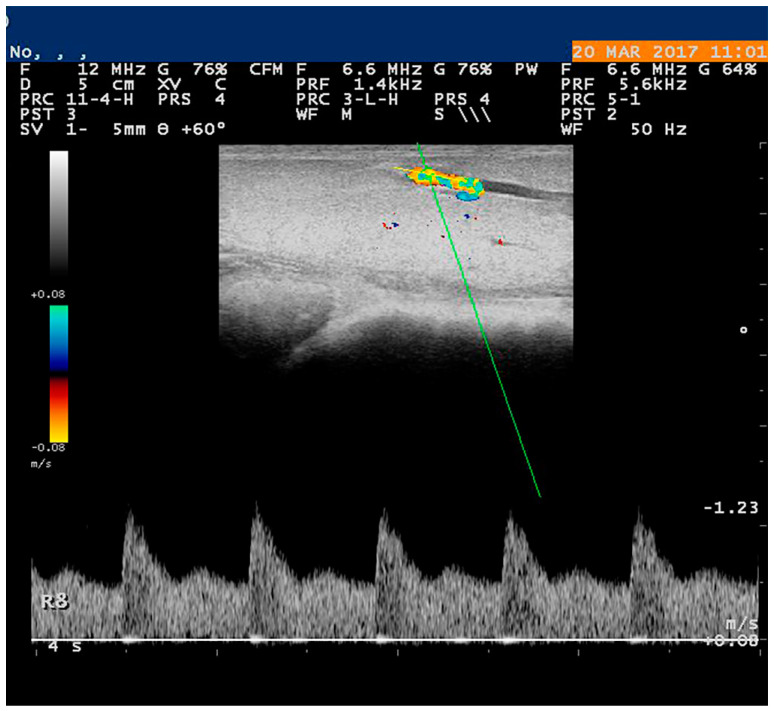
Spectral waveform of the external pudendal artery in the mammary gland of ewe. Image taken and processed on a MyLab^®^ 30 ultrasonography system with linear transducer 6.6 MHz and scanning depth 50 mm.

During the evaluation of vascularization by means of spectral Doppler, the settings of the equipment are of particular importance in reliable measurement. In particular, the angle between the Doppler beam and the vessel’s long axis (‘Doppler angle’) should not exceed 60°, colour gain must be adjusted in a way that colour visualizes only inside the vessel, and the Pulse Repetition Frequency should be set appropriately to avoid the ‘aliasing phenomenon’ [41]. The Doppler gate, which is dependent on the diametre of the vessel, should be regulated at 2 to 4 mm and positioned at the center of the vessel under examination (Figure 1). A minimum of three continuous and consecutive waves are needed for an accurate evaluation. The parametres measured in most studies are the following: (a) peak systolic velocity (PSV), (b) end diastolic velocity (EDV), (c) resistance index [RI = (PSV − EDV)/PSV], and (d) pulsatility index [PI = (PSV − EDV)/TAMV] (TAMV: time-averaged maximum velocity); in some cases, important information can also be extracted by (e) systolic: diastolic velocity ratio [SV/DV = (ASF/ADF)] (ASF: average diastolic flow and ADF: average systolic flow), (f) time-averaged maximum velocity (TAMV), (g) systolic acceleration (A), and (h) total blood volume [Q = (TAV_mea_n × S)] (TAV_mean_: time-averaged mean velocity, S: cross-sectional surface of the vessel) [41,42]. Finally, the characterization of blood flow pattern has also been reported, as the laminar or turbulent flow and the high or low resistivity are observations that may contribute to the diagnosis [43]. Potentially, the assessment of the blood flow pattern can be used to assess the efficacy of anti-vascular therapy [44,45].

During the examination, the farm animals should be in a standing position, restrained by an assistant [41] whilst the companion animals should be in lateral recumbency and no sedation during examination is needed [4]. The hair of the specific region should be fully clipped [41].

In farm animals, the technique has been applied in healthy animals for monitoring and evaluation of mammary blood flow in lactating [46] or dry [47] cows, in heifers [48], in ewes [49,50] and goats [51,52]. Regarding mastitis, many studies have employed the Doppler ultrasonographic examination for the investigation of the infection in cattle [53], buffaloes [54], and sheep [50], thus nowadays the Doppler examination is considered a precise method for the diagnosis of this disease. Santos et al. [55] have indicated that increased PSV and RI in cases of mastitis in goats are probably related to more severe infections. Furthermore, the examination of the supramammary lymph nodes may also provide useful information regarding infection in cows [42].

In companion animals, by using colour flow imaging, a mainly peripheral vascular pattern has been observed in benign tumours in dogs whilst in mammary malignant lesions, a mixed pattern has been usually reported [43,56]. Moreover, by using the spectral mode, increased blood flow has been observed in malignant lesions, specifically a significant increase in PSV [43] whilst no differences were detected in PI or RI [57]. The use of the modality in mastitis in dogs has been reported by Balaci et al. [58] and Trasch et al. [36], who used Doppler examination for evaluating the efficacy of treatment protocols against mastitis. It seems that Doppler ultrasound evaluations may assist in the prediction of the malignancy of canine mammary masses but with moderate sensitivity and specificity [5].

The advantages of the technique are the real-time information about vascularization and haemodynamic aspects of blood vessel flow that it can provide [59] and the affordable cost of the equipment [41]. The disadvantage may be the inability to detect small lesions or microcalcifications that may lead to false negative results in companion animal tumours [60].

### 5.2. Contrast-Enhanced Examination

**Figure 2:**
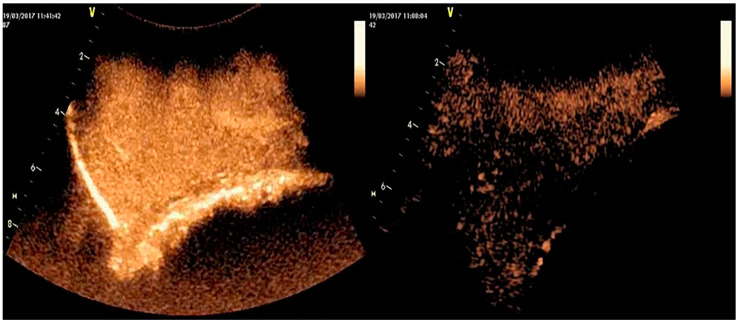
Contrast-enhanced ultrasonographic presentation of mammary parenchyma at the 6th month of the lactation period. Along the long axis of the udder, left: imaging of mammary gland of a healthy ewe, with peak enhancement of mammary parenchyma in 24 s—right: imaging of mammary gland of a ewe with history of mastitis, with reduced enhancement of mammary parenchyma in 46 s (images taken and processed on a Vivid-I ultrasonography system (General Electric) with convex transducer, imaging frequency: 2.0/4.0 MHz—mechanical index: 0.09—power: 22 dB—scanning depth: 60 mm—contrast agent: 20 µL sulphur hexafluoride in microbubbles) [62].

Contrast-enhanced ultrasonographic examination (CEUS) is a novel imaging technique that, in humans, is employed for the evaluation of mammary tumours [61]. Recently, CEUS has been found useful for the detection of lesions in mammary glands of animals [62]. For this examination, the ultrasound equipment must be fitted with specific software to perform the secondary harmonic imaging and inverted pulse whilst the use of a contrast agent is also necessary [62]. The microbubbles used as contrast agent must possess certain characteristics: stability with resistance to external pressures to prevent their dissolution and reduced gas diffusion into the blood [63]. Nowadays, the microbubbles used have a small size (1–10 mm in diametre); include a protein, lipid, or polymer shell capsule; and contain an inert and relatively insoluble gas [56,64,65]. Great attention must be paid to parametres like depth, gain, and focal zones, which must be maintained constant throughout the examination [5]. Video clips are taken immediately and for a short period after the injection of the contrast agent and recorded. Microbubble perfusion and the dynamic enhancement of the image of each lesion are thus analyzed, considering the presence or absence of the contrast agent in the tissue and the times of tissue perfusion from wash-in to enhancement peak to washout [5] (Figure 2).

During the performance of the technique, the contrast agent is administrated intravenously through the cephalic (dogs, cats) or the jugular (ruminants) vein [62,66].

In human medicine, as found in mouse animal model, the tumour growth time can be identified by using CEUS examination [61]. CEUS is applied for predicting the nature of mammary lesions, in that way reducing the number of biopsies performed in patients [67]. The increased correlation of lesion vascularization in mammary tumours found by using CEUS with the results of MRI, further highlights the significant accuracy of the method [68]. A recent variation of the classical CEUS is the intradermal and subcutaneous injection of contrast agent in the region of the mammary areola, which may be of clinical application in breast cancer patients [69]. Although there are many studies certifying the diagnostic efficacy of CEUS examination for the characterization of mammary lesions in humans [70,71], in animal health the technique has received limited attention. In farm animals, there is only one study [62]. This study indicated that the reduced perfusion of a contrast agent into the mammary parenchyma of ewes correlated with the reduced amount of functional mammary tissue in long-standing mammary lesions [62].

In companion animals, the method has been described in mammary glands and inguinal lymph nodes in healthy dogs at the various stages of the oestrous cycle [66] and also in dogs with mammary tumour [10]. Moreover, a transcutaneous injection of contrast agent has been applied in dogs’ mammary glands, which was used as an animal model [72,73]. Recently, Stan et al. [10] proposed the combination of B-mode, Doppler, and CEUS examinations along with real-time elastography for the assessment of sentinel lymph nodes in dogs with mammary carcinoma. They indicated that elastography had the highest accuracy in identifying metastases in sentinel lymph nodes whilst CEUS and Doppler examinations had similar accuracy. However, in another study, the use of CEUS in mammary lesions of dogs did not conclude to differentiation of neoplastic and non-neoplastic tissues [74].

The advantages of CEUS examination include the imaging of the progression of vessel formation and evaluation of blood flow in functional vessels [75], which is important information in the investigation of mammary tumours. The disadvantages may be the inability to detect small lesions, as in a study in human hepatocellular carcinoma where the smallest detectable lesions ranged from 3 to 5 mm in diameter [76], and the low specificity (16.7%) of the method [5].

### 5.3. Three-Dimensional Examination

Three-dimensional (3-D) ultrasonography is a relatively new imaging method that has received a limited application in mammary imaging in animals [77,78] despite its extensive use and rapid development in humans [79,80]. The three-dimensional ultrasonographic examination of the breast is a useful tool for various procedures, e.g., breast biopsy or anatomic guidance during breast neoplasia surgery [81,82].

For this technique, a conventional transducer with or without an electromagnetic position sensor can be used. However, in the latter case, defects may appear on the display, as, during movement of the transducer across the region of interest in order to capture multiple two-dimensional images, some areas may easily be under- or over-scanned and the slice spacing may vary; hence, the data corresponding to the volume of structures of interest would not be accurate if an electromagnetic position sensor was not used [83]. Therefore, nowadays, it is preferable to use the ‘volume transducer’, which is a modern transducer with a positioning system and scanning mechanics (a built-in mechanical drive to carry out a fully automatic sweep). The transducer is held and not moved whilst the transducer elements automatically sweep through the volume box, which is the operator-selected region of interest [77].

In a two-dimensional ultrasonographic examination, the target tissue is scanned in different planes and the operator must reconstruct a multitude of images in his mind in order to receive a virtual three-dimensional image [84]. In contrast, in the three-dimensional examination, image reconstruction is performed directly by the software of the equipment, and the processed signals are presented to the monitor as a real three-dimensional image [84,85].

In farm animals, there are only two bibliographic reports. In these, Fasulkov et al. [78] and Franz et al. [77] applied the technique for the visualization and characterization of structures of lactating mammary glands in healthy cattle and obtained clear imaging of the teat, the ductal system, and the mammary parenchyma. The same procedures in animals as in the Doppler examination are required for the performance of the 3-D examination [78].

In companion animals, the application of this technique has not been reported yet.

The advantage of this technique is the better sonographic discrimination of malignant from benign masses than 2D, as 3-D provides more information on vascular morphology [86]. The disadvantages could be the high cost of the necessary equipment and the need for specialized knowledge for applying the technique [87].

### 5.4. Elastography

Elastography is an ultrasonographic technique employed to measure the elasticity and stiffness of tissues, in order to increase diagnostic sensitivity [88]. The tissue elasticity is assessed by the evaluation of the pressure force applied on the tissue and the resulting deformation. The tissue deformation can be estimated by high-frequency echo signals, whilst a value of tension cannot be evaluated on the basis of the tissue measurement. In this respect, the procedure of exerting force on the tissues must be performed under strictly controlled conditions [89,90].

The elasticity of a tissue (even within the same organ) may vary according to the physiological state (e.g., lactating or dry mammary gland) or the pathological process (e.g., inflammation, neoplasia) in there. Hence, by using the appropriate software in the ultrasonographic equipment, this elasticity can be visualized [91]. During the examination, the equipment initially receives digitized radiofrequency echo signs from the tissue as it is (i.e., without compression); then, the compression applied to the tissue by the transducer along the radiation axis results in some displacement of the tissue; finally, the equipment receives a second, post-compression digitized radiofrequency echo sign from the same tissue [92]. The data received from these two echo lines (i.e., before and after compression) undergo processing, which results in the production of the ‘elastogram’ (i.e., the elastographic image).

Several types of elastographic techniques can be applied, which depend upon the force that is applied onto the tissue; these include real-time elastography, acoustic radiation force impulse imaging, transient elastography, point shear wave elastography, and shear wave elastography [93]. The determinant for the success of the technique is the force exerted by the ultrasound transducer on the tissue under examination [90]. Shear wave elastography is an advanced technique that allows objective measurement of the elasticity of a tissue [94,95]. In real-time elastography, the stiffness of the tissue is transposed in colour mode, where each colour signifies a level of stiffness: images with blue areas correspond to soft tissue areas, i.e., more elastic, whilst reddened areas correspond to more rigid tissue areas, i.e., with lower elasticity [5]. Then, the evaluation of shades of colours into scores of elasticities has been made possible [96,97].

In farm animals, the technique was described by Skerl et al. [94], who used samples of mammary glands of cattle to monitor the pressure applied during manually performed elastography.

In companion animals, acoustic radiation force impulse imaging (ARFI) has been used in dogs [5,98] or cats [99] with mammary tumours. Feliciano et al. [98] compared acoustic radiation force impulse results with carcinoma types and grades, as they were estimated by histopathological classification. They found that the method could identify complex carcinoma types with moderate accuracy due to the shorter periods of contrast wash-in and peak enhancement times observed in this type of tumour. They also found that the increase in perfusion times (wash-in, peak enhancement, and washout times) enables the detection of grade II and III carcinomas with moderate accuracy. Although the diagnostic technique is not sensitive enough for the differentiation of special and complex carcinoma types [98], it is highly recommended in veterinary oncology for malignancy prediction of canine mammary masses [5].

Additionally, shear wave elastography has been used for the differential diagnosis of benign from malignant lesions in canine mammary tumours [95,100]. Glińska-Suchocka et al. [95] performed shear wave elastographic examination in neoplastic mammary glands of dogs and then fine-needle aspiration biopsy to determine the nature and degree of malignancy of the lesions. They found that benign neoplasms of the mammary gland showed low stiffness, whereas malignant neoplasms were characterized by high stiffness. On the other hand, Pieczewska et al. [100] evaluated by shear wave elastography the density of the canine mammary tumour before and after the treatment with aglepristone and found no influence on the density of the tumour’s tissue. Among the various elastography techniques, shear wave elastography has been noted as the most promising tool, as it allows fast and non-invasive diagnosis of malignant mammary tumours in dogs [100].

Based on the above studies, it is inferred that elastography is a useful tool for predicting malignancy with high sensitivity (94.7%) and specificity (97.2%) [5], but, in many cases, the biopsy cannot be avoided [101].

## 6. Discussion

A variety of techniques can be used to visualize mammary lesions in the various available imaging techniques. In CT, x-ray radiation is used to receive a cross-sectional image of the body in MRI a magnetic field forces the hydrogen cellular nuclei to align in different positions, and in PET, the radiation emitted from the animal after injection of radioactive tracers is visualized [3]. In ultrasonographic examination, high-frequency sound waves are used with different modalities in order to receive tissue-related information [5]. Although imaging techniques vary, their use in animals depends on animal species and pathological condition. The mammary diseases that have been mainly studied in animals are mastitis and mammary tumours. Mastitis occurs frequently in cows and ewes and has a significant impact on animal’s welfare and a huge economical effect on the dairy industry [102,103], so its early diagnosis is imperative. On the other hand, mammary tumours have been studied thoroughly in dogs and cats. Mammary tumours are the most common neoplasm in intact female dogs [104] and the third most common neoplasm in domestic cats [105]. Therefore, the imaging of mammary tumours as well as the diagnostic accuracy of each method has concerned many researchers.

In human medicine, mammography and ultrasonography have an important role in the diagnosis of mammary diseases, but still, a biopsy guided with ultrasound examination is necessary for the histopathological confirmation of the findings [9]. MRI can provide additional information [106]. In addition, CEUS studies can reveal the presence of nodular-type lesions [9] while the combination of PET/CT may be useful in cases of mammary infections [9] and can be used to confirm mammary neoplasia [107,108,109]. Furthermore, Doppler examination can detect increased vascularization within the mammary gland [110] and also dilated lactiferous ducts with echoic content or with thickened walls, a finding that MRI can also reveal [106]. In regard to the mammary neoplasia, Doppler examination has been used for the quantitative evaluation of the perfusion of the mammary parenchyma and the vascularization of neoplastic tissues [111,112], as well as for the differentiation between malignant and benign mammary lesions [113]. It seems that sometimes, imaging features cannot provide differentiation between mammary neoplasia or infection; for example, ultrasonographic examination would show irregular hypoechoic masses with increased vascularity while MRI would show irregular, enhancing masses or non-mass enhancement with micro-abscesses [114]. Although in human medicine diagnostic imaging guidelines have been proposed to investigate diseases of the mammary glands [9], in veterinary work, similar protocols have not been developed thus far and only an algorithm has been proposed for the evaluation of sentinel lymph nodes in dogs with mammary carcinoma [10].

In farm animals, the ultrasonographic examination of the mammary glands has been studied extensively [37,41,50] while the rest of the imaging methods were studied less, mainly for the estimation of the normal parenchyma [24,31] or for highlighting the benefits of the methods in human medicine when animal model studies were performed [115]. The easy transfer of the ultrasound machine to the farm, the fast real-time imaging, and its application without the need for anesthetizing the animal [41] are reasons for its wide use (Table 1). Ultrasonography has been proposed as a diagnostic technique for the diagnosis of subclinical mastitis in sheep [116] and as a tool indicating the proper time to start treatment [117] and also monitoring the progress of mastitis in cows [118]. After the ultrasonographic examination, microbiological examination and California mastitis test (CMT) of milk samples are employed to confirm the disease [119].

Nowadays, in clinical practice, mammary neoplasia in dogs and cats is usually detected by radiography, ultrasound, or computed tomography and, subsequently, is confirmed with histopathological examination [2,120,121]. For the differentiation of mammary malignant and benign tumours, Doppler examination (colour flow and spectral mode) is useful [10] whilst contrast-enhanced ultrasonography and acoustic radiation force impulse imaging elastography can be applied for the identification of some of the characteristics of mammary carcinoma in dogs [98]. Feliciano et al. [5] recommended the use of acoustic radiation imaging elastography in veterinary oncology, as it allows the fast, non-invasive, and complication-free prediction of malignant tumours in dogs. On the other hand, Gasser et al. [74] attempted to reveal differences between different types of benign mammary lesions in dogs by using Doppler, acoustic radiation imaging elastography, and contrast-enhanced techniques, but no conclusive findings could be obtained. Additionally, in animals with mammary tumours, the examination of lymph nodes and detection of potential metastases is particularly important, as well as the detection of lung metastasis, where the CT has been found to have higher sensitivity than chest radiographies [2]. In cases of metastatic lymph nodes, acoustic radiation imaging-elastography is the ideal method that may differentiate free and reactive tumour metastatic lymph nodes [122]. Contrast-enhanced ultrasound has improved the diagnostic possibilities regarding mammary abnormalities, as contributes to malignancy prediction [123]. Last but not least, MRI and PET are promising methodologies that have been applied to companion animals [4,8]; however, high equipment costs limit their application in clinical practice [3,4].

In conclusion, concerning mastitis in farm animals, ultrasonographic examination (Doppler, CEUS, elastography) could be performed as a first examination for the diagnosis of clinical or subclinical mastitis and treatment guidance, but a bacteriological examination and a California mastitis test (CMT) of milk samples should follow for the confirmation of the disease [116]. Regarding mammary tumours in companion animals, a combination of imaging techniques should be applied for accurate diagnosis. Initially, a first assessment of the extent of the tumour, its vascularity, and its characterization could be carried out using Doppler ultrasonography [124], and then, depending on the available equipment, CT, MRI, or PET can be applied, taking into account that CT and MRI provide information about anatomy while PET about the functionality of the tissue. Whichever imaging modality is used, biopsy or ultrasound-guided biopsy in cases of small sized not palpated lesions for histopathological examination is essential for an accurate diagnosis [125].

## Figures and Tables

**Table 1:** Diagnostic imaging methods applied to animals.

Method	Main Target	Preparation	Equipment	Cost	Species
Computed Tomography [12,14,15,17]	Mammary tumours and metastatic lymph nodes	Under general anaesthesia Optional administration of contrast agent	Not portable	++	Companion animals
Positron Emission Tomography [3,8,24]	Mammary tumours and metastatic lymph nodes	Under general anaesthesia Administration of radioactive isotopes	Not portable	++++	Companion animals
Magnetic Resonance Imaging [3,4,31]	Mammary tumours	Under general anaesthesia Optional administration of contrast agent	Not portable	+++	Companion animals
Doppler Examination [41,43,53]	Mammary tumours Mastitis	The hair of the region fully clipped	Portable	+	Companion animals (tumours) Farm animals (mastitis)
Contrast-enhanced ultrasonographic examination (CEUS) [62,66,10]	Mammary tumours Mastitis	The hair of the region fully clipped Administration of contrast agent	Portable	++	Companion animals (tumours) Farm animals (mastitis)
Three-Dimensional [77,78,87]	No report in mammary diseases	The hair of the region fully clipped	Portable	+++	Farm animals (healthy)
Elastography [5,99,100]	Mammary tumours	The hair of the region fully clipped	Portable	++	Companion animals (tumours)

## Data Availability

Not applicable.
